# Targeting cyclin D1 for mantle cell lymphoma

**DOI:** 10.1186/2051-1426-3-S2-P430

**Published:** 2015-11-04

**Authors:** Jingtao Chen

**Affiliations:** 1 Institute of Translational Medicine, The First Hospital, Jilin University, Changchun, Jilin, China

## 

Cyclin D1, an important component of cell cycle and a protein with known oncogenic potential, is over expressed in mantle cell lymphoma (MCL). MCL is a distinct clinical pathologic subtype of B cell non-Hodgkin's lymphoma often associated with poor prognosis. New therapeutic approaches based on boosting anti-tumor immunity are being developed. Targeting cyclin D1 for MCL is rendering an interesting target for immunotherapy. However, the knowledge on the frequency and profile of cyclin D1-specific T cells in MCL patients is fragmented. Here we show that both healthy individuals and MCL patients have a broad repertoire of cyclin D1-specific CD4^+^ and CD8^+^ T cells covering numerous epitopes from the whole cyclin D1 protein. Cyclin D1-specific T cells secrete IFN-g and type 2 cytokines including IL-5 and IL-13. Additionally, DCs loaded with whole tumor cells or with selected peptides can elicit cyclin D1-specific CD8^+^ T cells that kill MCL tumors. Furthermore, a recombinant vaccine based on targeting cyclin D1 antigen to human DCs via an anti-CD40 mAb was developed. Targeting monocyte-derived human DCs in vitro with anti-CD40-cyclin D1 fusion protein expanded a broad repertoire of cyclin D1-specific CD4^+^ and CD8^+^ T cells. Therefore, cyclin D1 represents a good target for immunotherapy.

